# Design, characterization and in vivo functioning of a light-dependent histidine protein kinase in the yeast *Saccharomyces cerevisiae*

**DOI:** 10.1186/s13568-018-0582-7

**Published:** 2018-04-02

**Authors:** Aleksandra Bury, Klaas J. Hellingwerf

**Affiliations:** Molecular Microbial Physiology Group, Swammerdam Institute for Life Sciences, University of Amsterdam, Science Park 904, 1098 XH Amsterdam, The Netherlands

**Keywords:** YtvA, Cytoplasmic diffusion, Sln1, Wall stress, Two-component regulation system, Nuclear shuttling

## Abstract

Helical alignment of the α-helical linker of the LOV (light-oxygen-voltage) domain of YtvA from *Bacillus subtilis* with the α-helical linker of the histidine-protein kinase domain of the Sln1 kinase of the phospho-relay system for osmoregulation of *Saccharomyces cerevisiae* has been used to construct a light-modulatable histidine protein kinase. In vitro, illumination with blue light inhibits both the ATP-dependent phosphorylation of this hybrid kinase, as well as the phosphoryl transfer to Ypd1, the phosphoryl transfer domain of the Sln1 system. The helical alignment was carried out with conservation of the complete Jα helix of YtvA, as well as of the phosphorylatable histidine residue of the Sln1 kinase, with conservation of the hepta-helical motive of coiled-coil structures, recognizable in the helices of the two separate, constituent, proteins. Introduction of the gene encoding this hybrid histidine protein kinase into cells of *S. cerevisiae* in which the endogenous Sln1 kinase had been deleted, allowed us to modulate gene expression in the yeast cells with (blue) light. This was first demonstrated via the light-induced alteration of the expression level of the mannosyl-transferase OCH1, via a translational-fusion approach. As expected, illumination decreased the expression level of OCH1; the steady state decrease in saturating levels of blue light was about 40%. To visualize the in vivo functionality of this light-dependent regulation system, we fused the green fluorescent protein (GFP) to another regulatory protein, HOG1, which is also responsive to the Sln1 kinase. HOG1 is phosphorylated by the MAP-kinase-kinase Pbs2, which in turn is under control of the Sln1 kinase, via the phosphoryl transfer domain Ypd1. Fluorescence microscopy was used to show that illumination of cells that contained the combination of the hybrid kinase and the HOG1::GFP fusion protein, led to a persistent increase in the level of nuclear accumulation of HOG1, in contrast to salt stress, which—as expected—showed the well-characterized transient response. The system described in this study will be valuable in future studies on the role of cytoplasmic diffusion in signal transduction in eukaryotic cells.

**Electronic supplementary material:**

The online version of this article (10.1186/s13568-018-0582-7) contains supplementary material, which is available to authorized users.

## Introduction

During the last decade of the previous century, progress in the dynamic resolution of protein structure, in the availability of genomic DNA sequence information, and in the synthetic biology of the heterologous production of complex holo-proteins, have brought our understanding of the molecular basis of cellular signal transduction networks down to the atomic level (see e.g. (Ridge et al. 2003)). This development was aided by the modular nature of many signal transduction proteins, which is particularly notable in the dominant type of prokaryotic signal transduction network, the ‘two-component regulatory system’, including its more complex variant, the ‘phosphorelay system’ (Nixon et al. 1986; Burbulys et al. 1991). In this development photosensory receptor proteins did play an important role because of the ease and accuracy with which these proteins can be (de)activated (for review see e.g. (van der Horst and Hellingwerf 2004; Hoff et al. 1997)). Understanding of the atomic basis of the structural and dynamic aspects of the transitions between the receptor- and the signalling state of signal transduction proteins then led to the development of rational and intuitive guidelines to combine functional (input/output) domains into new functional chimera’s, as could be concluded from analyses of their performance both in vitro and in vivo (Levskaya et al. 2005; Wu et al. 2009; Möglich et al. 2009).

These technical developments, and the derived improved insight, have led to the emergence of the interdisciplinary research field of ‘optogenetics’ (Miller 2006; Ernst et al. 2008; Zhang et al. 2010). This field meanwhile has made radically new and very important contributions to the disciplines of both cell biology (Bacchus and Fussenegger 2012) and neurobiology (Kim et al. 2017). Gradually, these developments now also start to impregnate the field of biotechnology, including the area of sustainability applications of ‘direct conversion’ (Savakis and Hellingwerf 2015) with cyanobacteria (Abe et al. 2014; Miyake et al. 2014).

Complete understanding of cellular signal transduction networks, however, not only requires understanding of the dynamics of the structural transitions within the protein components involved, but—particularly for those operating in the larger, i.e. mostly eukaryotic, cells—also resolution of the spatial dimension of such processes. This latter aspect is not only dictated by association/dissociation kinetics of the underlying physicochemical signals (e.g. an electric field or osmotic pressure), signaling molecules and signal-transmission- and output proteins, but also by the processes of classical- and/or anomalous diffusion of all these components, either in the cytoplasm or in the cytoplasmic membrane, with possibly additional effects of molecular crowding.

To resolve (part of) these latter aspects, it would be of great value to have a signal-transduction system available that can be triggered with (a flash of) visible light, and that initiates relocation of a specific component of that signal transduction network in the cell, like e.g. between subcellular compartments. Here we report the design, construction and in vitro and in vivo testing of such a network. Our approach is based on the construction of a chimeric histidine protein kinase, composed of the light-oxygen-voltage, LOV, domain of the stressosome protein YtvA from *Bacillus subtilis* (van der Steen et al. 2012) as the signal input domain and the histidine-protein kinase domain of the Sln1 kinase (Li et al. 2002) of a two-component regulatory system of the yeast *Saccharomyces cerevisiae* as the output domain, for relay of the (light) signal to the downstream components.

**Fig. 1:**
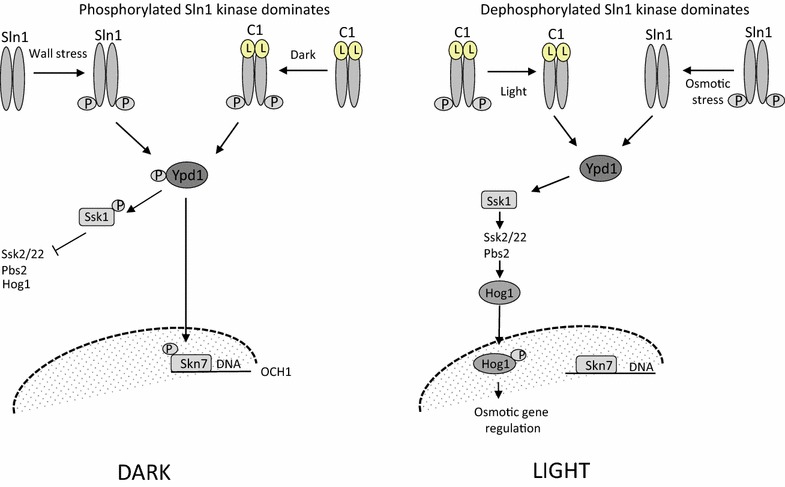
Schematic drawing of the flow of phosphoryl groups through the Sln1 pathway of *S. cerevisiae* in the wild-type version and with a LOV-histidine kinase fusion protein replacing the Sln1 kinase. The left-hand panel shows the flow of phosphoryl groups under wall-stress conditions (maximal activity of native Sln1) and in the dark (maximal activity of the C1, fusion protein). The right-hand panel shows the absence of flow of the phosphoryl groups in the presence of osmotic stress (for the native Sln1) and upon illumination (for the C1 fusion protein). The C1 fusion protein is most active in the dark. This leads to phosphorylation of the Ssk1 and Skn7 response regulators via transfer of the phosphoryl group through the Ypd1 phospho-transfer protein. Phosphorylation of Ssk1 renders it inactive in its role in the HOG1 pathway. Phosphorylation of Skn7 leads to activation of Skn7-dependent genes, such as the mannosyl-transferase, *OCH1*. Osmotic stress and illumination lead to reduction in the kinase activity of Sln1 and the C1 fusion protein, respectively, and therefore accumulation of the kinase domain of Sln1 in the dephosphorylated form. This causes de-phosphorylation of the Ssk1 response regulator, which can interact with and activate Ssk2 and the Ssk22 kinases of the HOG pathway. Phosphorylation of HOG1 leads to its translocation into nucleus and activation of the genes responsible for osmotic-stress regulation (Fassler and West 2010; Gao and Stock 2009)

The Sln1 kinase of *S. cerevisiae* is part of the ‘wall stress’ signal transduction network of this yeast (for a brief overview: see Fig. 1) and has the typical structure of a phospho-relay system (Gao and Stock 2009; Fassler and West 2010). Its input kinase is located in the cytoplasmic membrane of yeast cells and able to convert signals derived from damage of components of their cell wall and of (a) signal(s) derived from osmotic stress, into changes in the level of phosphorylation of the cytoplasmic phosphoryl transfer domain, Ypd1 (Ferrigno et al. 1998). The level of phosphorylation of Ypd1 modulates nuclear gene expression directly (e.g. of Skn7), and also indirectly—via the MAP kinase pathway of the Ssk system—through the shuttling of the transcriptional regulator HOG1 between the cytoplasmic and nuclear compartment (Lu et al. 2003). Via analysis of the spatial distribution of fluorescent reporters in fixed *Saccharomyces* cells, sampled after triggering of either the natural- or an engineered LOV::Sln1-containing signal transduction network, we have been able to show the functionality of the designed chimeric light-dependent histidine protein kinase.

## Materials and methods

### Growth of *S. cerevisiae*

The starting strain ∆YLR113W (Additional file 1: Table S2) was cultivated on rich medium (YPD) agar plates, followed by growth in YPD liquid medium, at 30 °C in a shaking incubator, followed by growth on minimal complete medium for gene knock out and plasmid transformation experiments. For selection of the Sln1 knockout strain, nourseothricin sulphate (clonNAT) was added to the minimal complete medium. To select for the continued presence of the plasmids, carrying the required customized version of the genes constructed, minimal drop out media were used.

### Molecular genetics and protein purification

Genes encoding a required sequence (e.g. of a hybrid kinase) were amplified via PCR and cloned into a pQE vector (Qiagen, Hilden, Duitsland) for heterologous overexpression as a poly-histidine tagged protein in *E. coli,* and in a pRSII (Chee and Haase 2012) vector for in vivo expression in *S. cerevisiae*. DNA-fusion constructs were generated by overlap-extension PCR. After gene expression in *E. coli*, the recombinant proteins were purified from the cell-free extracts in a two-step procedure that makes use of: (i) Affinity chromatography on a HisTrap FF column (GE Healthcare, Chicago, Illinois, United States, 5 mL column) and (ii) Anion exchange chromatography on a ResQ column (GE Healthcare, Chicago, Illinois, United States 6 ml column volume). For all proteins containing the LOV domain, their concentration was determined using the extinction coefficient of 14,000 M^−1^ cm^−1^ at 450 nm (Koziol 1971). The Bradford method was used for all other proteins (Bradford 1976) (see also Additional file 1: Tables S1 and S2).

### In vitro assay of the extent and rate of phosphorylation of the hybrid histidine protein kinases and of phosphoryl transfer from Sln1 kinase- to the Ypd1 phosphoryl transfer domain

Kinase activity assays were carried out after slight modification of established procedures (Fassler and West 2010): 30 µM of the specific histidine protein kinase was incubated with 1–5 mM cold ATP, after mixing of the latter with 3300 Ci/mmol of [γ-^32^P]-ATP. All reactions were carried out in a buffer containing 50 mM Tris∙HCl pH = 8, 100 mM KCl, 15 mM MgCl_2_, 2 mM DTT and 20% (v/v) glycerol in a total volume of 0.1–1 ml in Eppendorf tubes. Time-series samples were taken between 0 and 30 min at regular intervals. Samples were immediately mixed with 33% (v/v) fourfold concentrated stop buffer. This concentrated stop buffer contains 0.25 M Tris∙HCl pH = 8, 8% (w/v) SDS, 40% (v/v) glycerol, 40 mM EDTA, 0.008% (w/v) bromophenol blue and 4 mM β-mercapto-ethanol. For the phosphoryl transfer experiments, the histidine kinases were first autophosphorylated for 1 h in the dark, after which Ypd1 was added to the sample in a molar ratio of 1:2. Time-series samples were taken between 1 and 30 min. Samples were immediately mixed with stop buffer, just as described above. Samples were analyzed on 10% (w/v) SDS PAGE gels, which were then exposed to a GE Healthcare screen (GE Healthcare, Chicago, Illinois, United States). Screens were scanned with a Typhoon Fla 7000 system (GE Healthcare, Chicago, Illinois, United States) and the resulting data files were saved as.tiff files. Image Quant software (GE Healthcare, Chicago, Illinois, United States) was used for the quantification of the intensity of the different bands. Kinase phosphorylation experiments and phosphoryl transfer experiments were conducted in the dark, with minimal red background light (Avila-Perez et al. 2006), or under constant illumination from blue light emitting diodes (LEDs with λ^max^ = 464 nm) with an incident light intensity of 200 µEinstein m^2^ s^−1^.

### Activation of the Sln1 kinase domain in vivo with (stress) signals

For the application of the osmotic stress signal, 0.4 M NaCl (final concentration) was added to 2 ml cell suspension, growing exponentially in minimal selection medium, at 30 °C, on a rotary shaker in 12 ml glass tubes (Fassler and West 2010). Light activation of the hybrid LOV-kinase protein was achieved with blue LEDs with λ^max^ = 464 nm, with an incident light intensity of 200 µEinstein m^2^ s^−1^. Cells were fixed with 0.37% (v/v) *p*-formaldehyde and rapidly frozen in liquid nitrogen for further analysis (Fassler and West 2010).

### Measurement of the level of expression of reporter enzyme via β-galactosidase activity

Overnight cultures of recombinant strains of *S. cerevisiae* were grown in yeast extract peptone dextrose (YPD) medium in the dark, starting from a single colony from a plate of the minimal selection medium. The overnight cultures were diluted to OD_600_ = 0.05 and allowed to grow in the dark or in the light for 6 h. Dark cultures had been wrapped tightly in tinfoil. Dark samples were taken with minimal red background light intensity (Avila-Perez et al. 2006; see above). Samples from illuminated cultures were taken under constant illumination with blue LEDs (λ^max^ = 464 nm) with an incident intensity of 200 µEinstein m^2^ s^−1^. Samples were immediately transferred to an ice/water mixture and immediately flash frozen with liquid nitrogen for subsequent storage at − 80 °C. β-galactosidase activity was measured in the cells from all samples and expressed in Miller units, based on the average value of at least 8 independently isolated transformants (Miller 1972).

### Microscopy

Log-phase cultures of the yeast *S. cerevisiae*, expressing a HOG1::GFP (green fluorescent protein) fusion protein, were fixed with 0.37% (v/v) *p*-formaldehyde for 1 h, washed, re-suspended in phosphate-buffered saline (PBS) pH = 7, and stained with 0.5 µg 4',6-diamidino-2-phenylindole (DAPI) per ml culture to visualize the nuclei of the cells. The yeast cells were observed using a Nikon Eclipse Ti inverted microscope (Shinagawa, Tokyo, Japan), equipped with a 100× objective. Fluorescence emission signals of GFP and DAPI were generated using a Lumencor (Beaverton, United States) fluorescent light source and detected at 470 and 395 nm, respectively. Images were captured using a Hamamatsu digital camera C11440 (Hamamatsu City, Japan) driven by the Nikon elements AR 4.50.001 software (Shinagawa, Tokyo, Japan). All pictures of cells with a specific fluorophore were acquired using the same exposure time: 100 ms for DAPI and 400 ms for GFP. The pictures were then analyzed using ImageJ software (Schindelin et al. 2012) without further manipulation. For analysis, images were exported as.tiff files for import into ImageJ software. For quantitative analysis of the microscopy data, pictures of cells with DAPI-stained nuclei, and with HOG1::GFP expression, were overlapped. Cells with nuclear- and cytoplasmic localization (only cells with > 1.5-fold nuclear accumulation were counted as positive; by definition the others as cells with cytoplasmic localization) of the HOG1::GFP reporter protein were counted and the percentage of cells with nuclear localization was calculated and plotted.

## Results

### In vitro phosphorylation assays

**Fig. 2:**
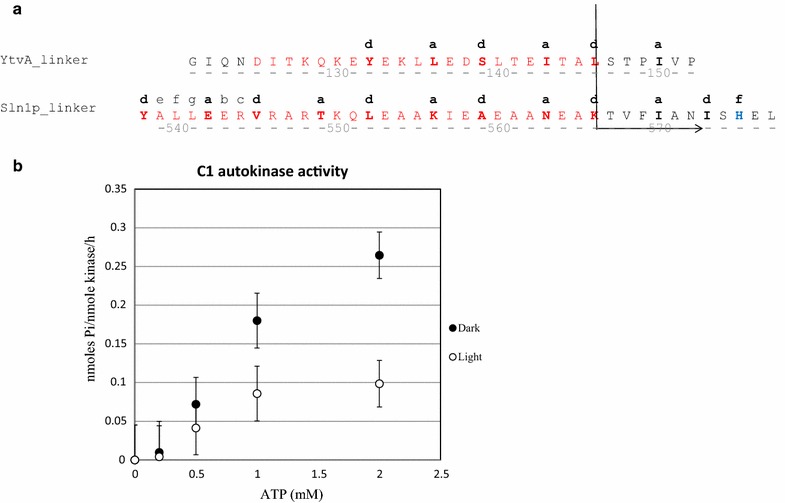
**a** Alignment of the coiled coil structures from Sln1 and YtvA. The point of switching over from the amino acid sequence of the Jα helix of YtvA to the Sln1 sequence in the C1 construct is indicated by the arrow. **b** Effect of light on the incorporation of [^32^Pi] into the C1 histidine kinase fusion protein. [γ-^32^P]-ATP was used as the phosphoryl group donor. Reaction mixtures were incubated either in the presence of light (open symbols) or in the dark (filled circles). Error bars represent the standard deviation calculated from three independent experiments. For further detail: see Materials and methods

The helical linker regions of YtvA and Sln1 were aligned according to the hepta-helical pattern of the coiled-coil structure that presumably is present in both of them, and joined in several different ways (Additional file 1: Table S1 and Fig. 2a), i.e. with preservation of the (Jα-)helix from either protein completely, or partially; with or without insertion of extra amino acids to translationally shift the hepta-helical pattern and with or without conservation of the position of the crucial phosphorylatable histidine of the Sln1 kinase domain. The resulting hybrid kinases, with the truncated Sln1 kinase domain as a reference, were assayed for kinase activity in the dark with the classical kinase assay based on the use of ^32^P[ATP]. Other assays, e.g. based on inorganic phosphate release, were tested too, but turned out to be less suited.

Of the initial series of hybrid kinases tested (i.e. C1 to C8), only the truncated reference domain and the C1, C2 and C6 fusion proteins showed considerable auto-phosphorylation activity, in the order of 0.38, 0.24 and 0.24 nmolP/g protein/min for the latter three, respectively, at saturating concentrations of the nucleotide substrate (i.e. 5 mM; see (Fassler and West 2010)) and 30 µM of the specific histidine protein kinase (domain). Next, we tested a possible difference between this activity, and the corresponding activity in saturating intensities of blue light (for further experimental detail: see Materials and methods). These assays revealed that significant differences in activity, when assayed in light and dark, were only observed for the fusion protein C1 (while a very small difference was observed for C6; see Additional file 1: Table S1). The former, i.e. C1, in spite of its lower maximal activity, was therefore selected for further experiments. Significantly, for both hybrid kinases it turned out that illumination lowered their activity. In subsequently designed fusion proteins (e.g. C11) it turned out to be possible to observe significant light-stimulation of kinase activity (Additional file 1: Table S1). As for our subsequent in vivo experiments (see below) the light-inhibition of kinase activity was most valuable, these latter constructs have not been further characterized.

For the C1 light-modulatable histidine protein kinase we then characterized the kinetic basis of its light sensitivity. Time-course phosphorylation experiments in a time window of 120 min revealed that in most experiments the increase in the degree of phosphorylation of the kinase was approximately proportional with time during the first 30 min (Additional file 1: Fig. S1). Therefore, the dependency of the rate of autophosphorylation of the C1 kinase on the concentration of ATP was investigated with a range of nucleotide concentrations from 0 to 5 mM (Fig. 2b). These experiments revealed that under both assay conditions (i.e. in light and in the dark) the half-maximal rate of phosphorylation is observed at about 0.5 mM ATP, while the maximal rate of phosphorylation (V_max_) is lowered with more than 50% in the presence of saturating amounts of blue light (Fig. 2b). We do not refer to Km values here because under both conditions the rate of phosphorylation appears to be dependent on the nucleotide concentration in a slightly sigmoidal way, which may be due to allosteric regulation of the kinase activity. This latter point, however, was not further investigated.

Besides their autophosphorylation activity, several of the hybrid kinases were also tested for activity in an assay that measures phosphoryl transfer from the kinase/response-regulator domain of Sln1 to the phosphoryl-transfer domain of this phosphorelay system, i.e. Ypd1. All constructs except C9 were active in this assay; (data not shown) however, because we did not have a rapid-quench system available (compare (Janiak-Spens et al. 2005; Kaserer et al. 2010)), we could not time-resolve this process, and therefore not differentiate between the different hybrid kinases with respect to this activity.

### In vivo functionality of the hybrid kinase in the Skn7 signal transduction pathway

**Fig. 3:**
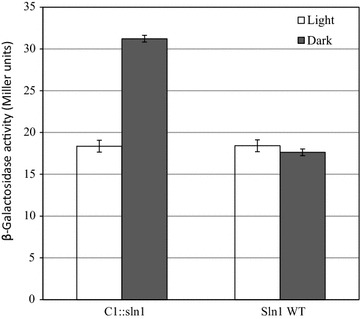
Effect of illumination on the Skn7-dependent expression of the *OCH1*-*lacZ* transcriptional fusion (i.e. the mannosyl-transferase gene fused with the reading frame encoding β-galactosidase) in a *S. cerevisiae* strain with the wild-type sln1 gene, and in a strain with sln1 replaced by the gene encoding the C1-histidine kinase fusion protein. Cells were incubated either in the presence of light (white bars) or in the dark (grey bars). The level of expression of the *OCH1*-*lacZ* fusion is deduced from the specific β-galactosidase activity, expressed in Miller units. Error bars indicate standard deviations calculated from three independent biological experiments

As outlined in the Introduction, the Sln1 phospho-relay system has two output pathways that target the HOG1 and the Skn7 nuclear transcriptional regulator, respectively. The Skn7 pathway is the most direct one of these two because Ypd1 directly phosphorylates Skn7 (Lu et al. 2003). We therefore first tested whether or not illumination, via the hybrid kinase C1, could elicit changes in the activity of Skn7. A suitable read-out of the latter is the level of expression of the mannosyl-transferase OCH1, via the reporter enzyme β-galactosidase, translationally fused to the former (Lu et al. 2003; Li et al. 2002). The results summarized in Fig. 3 clearly show that this is indeed the case: Switching on saturating intensities of blue light decreases this expression level about twofold and a decrease is indeed expected as illumination decreases the rate of phosphorylation of the C1 kinase. It is of note that the replacement of the native Sln1 kinase by C1 does give a doubling of the level of OCH1 expression, but this is presumably due to the deregulated expression and/or activity of the truncated kinase domain. The additional control experiment of illuminating wild type cells, clearly shows that without the introduction of the fused LOV domain-containing protein in this assay *S. cerevisiae* does not respond to light (Fig. 3).

### In vivo functionality of the hybrid C1 kinase in the HOG1 pathway: observation of light-induced nuclear shuttling

**Fig. 4:**
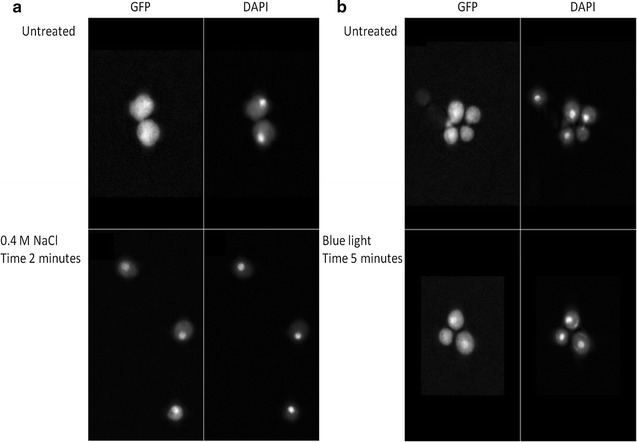
Subcellular localization of the HOG1::GFP fusion protein in response to changes in osmotic pressure (**a**), and after illumination (**b**) of the cells with blue light. Activation of Sln1 signaling was initiated with: **a** a change in osmotic pressure elicited by addition of 0.4 M NaCl (final concentration) to the cell suspension, and **b** illumination by exposure of the cells to blue light (200 μE incident intensity, 450 nm LED light). The strain used for **a** was: ∆HOG1, pRS416-HOG1::GFP; and for **b**: ∆HOG1, pRS416-HOG1::GFP, ∆Sln1, pRS325ActC1LEU

**Fig. 5:**
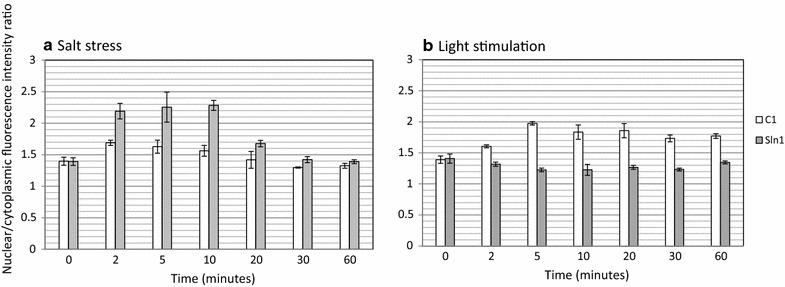
Nucleo-cytoplasmic redistribution of the HOG1::GFP fusion protein after a salt stress (**a**) and after illumination of the cells (**b**). The salt stress (**a**) was applied by addition of 0.4 M NaCl (final concentration) to the cell suspension. In **b** the results are shown of exposure of the cell suspension to blue light (200 μE incident intensity, 450 nm LED light). Samples were taken 0, 2, 5, 10, 20, 30 and 60 min after initiation of the experiment. White bars represent the yeast strain with the C1 histidine kinase fusion protein, and grey bars represent the yeast strain with the native Sln1 histidine kinase. Error bars represent the standard deviation calculated from three independent experiments

For this test we used two *S. cerevisiae* strains in which the endogenous Sln1 kinase and the HOG1 regulator protein had been genetically deleted and replaced by the C1 hybrid kinase and a translational HOG1::GFP fusion protein, respectively. The first one of these two strains had only the HOG1 gene replaced, and the second strain, both genes. With the resulting two strains stimulus/response experiments were carried out: With the first strain by eliciting an osmostress response, and in the second strain, which now is insensitive to osmostress, the occurrence of a light-response was tested (Figs. 4, 5). Through fluorescence microscopy of glutaraldehyde-fixed cells at emission and excitation wavelengths suitable for the analysis of their GFP- and DAPI content, respectively, we then analyzed the subcellular distribution of these two fluorophores, in which of course DAPI reveals the presence of the nuclear compartment, while GFP is present in both the nucleus and the cytosol. Figure 4a then shows the well-known response of the HOG1 protein in *S. cerevisiae* upon osmostress (Posas et al. 1996): An almost equal distribution of the HOG1::GFP fusion protein over the two compartments prior to the stress, followed by a rapid (i.e. within a few minutes) and significant accumulation in the nucleus after this stress. Panel b of Fig. 4 shows that the same response, i.e. HOG1 accumulation in the nucleus, can be elicited by exposing the yeast cells to saturating intensities of blue light of the yeast strain in which next to HOG1, also the Sln1 kinase has been eliminated and replaced by the C1 hybrid kinase. In Fig. 5a quantitative analysis of the dynamics of these two responses (i.e. to osmostress and to illumination) is presented. The osmostress response shows the typical transient response with maximally almost twofold accumulation in the nucleus of the HOG1::GFP fusion protein after around 5 min, and a full relaxation of this concentration gradient at long timescales (e.g. 30 min; compare ref (Posas et al. 1996)). The light-induced response in the strain carrying the hybrid C1 kinase, in contrast, shows the expected persistent response of a light-activatable system in continuous light, but appears to take more time to develop. More detailed analysis, at the level of the individual cells (Additional file 1: Fig. S2) shows that the nuclear accumulation of the fluorescent reporter (i.e. HOG1) in selected cells can increase up to fourfold (with salt stress) and slightly less (i.e. up to threefold) with illumination. Consistent with the results displayed in Fig. 5, also in Additional file 1: Fig. S2 we see the same slower kinetics with light activation. An additionally significant effect visible from this figure is the fact that the functionality of both signal transduction systems depends on the level of expression of the fluorescently labelled HOG1 protein: If the expression level of this protein is increased more than fivefold over minimum expression levels, neither the light- nor and stress-induced nuclear accumulation are detectable anymore.

## Discussion

### Design and activity of the LOV::Sln1 histidine kinase fusion proteins

The light-stimulated fusion proteins described in this study were designed following work reported by the group of Moffat, Möglich and co-workers (Möglich et al. 2009; Möglich and Moffat 2007, 2010). Their approach is based on the identification of the boundaries of the independently folded domains in signal-transduction proteins like FixL, YtvA, etc., and of the helices linking them. These linker helices often form a coiled/coil tertiary structure in dimers of the corresponding signal transduction proteins. Coiled/coil structures, besides their α-helical nature, display a seven- (hepta-) amino acid repeat structure, with a hydrophobic side chain at each 4th- and 7th-position (Diensthuber et al. 2013). This repeat structure then provides a rationale for domain swapping to make new functionalities via fusion proteins. A light stimulated histidine kinase can for instance be constructed by swapping sequences within these linker domains so that the stability of the independently folded domains would not be affected by the swap. Therefore, the coiled/coil linker helices, identified in YtvA and in Sln1, were aligned on the basis of the hepta-helical repeat motif that is identifiable in both of them (Fig. 2a) (Tao et al. 2002; Möglich et al. 2009). This alignment shows that the YtvA sequence, (directly) following the conserved DIT motif (amino acids # 125–127, which are key to signal transduction within the YtvA protein (for review: see (van der Steen et al. 2012)) can be fused with a non-cognate kinase domain. The Sln1 kinase domain qualifies for this in the region just upstream the phosphorylatable histidine of the kinase (i.e. amino acids # 512–540).

Based on the above considerations we designed the C1 construct in which the upstream sequences, including the linker helix of Sln1 are replaced by the LOV domain plus Jα helix, from YtvA. Constructs C2, C5 and C6 instead have the LOV domain fused to the Sln1 kinase domain directly after the conserved DIT motive. They differ among each other in the length of the helical linker of the Sln1 domain (see Additional file 1: Table S1) which will have an influence on the total length of the coiled/coil structure. In the C8 construct the part contributed by YtvA has been extended with 6 amino acids, as an attempt to enhance the difference in kinase activity between light and dark, following Mӧglich’s design of the YF2 construct (Möglich et al. 2009).

As in the C1 construct light-inhibition of kinase activity was observed, we also tried to design constructs with light-stimulated kinase activity. Such light-stimulated kinase activity was e.g. reported for the YF constructs described in Mӧglich et al. (Möglich et al. 2009). Their YF1 construct is composed of the LOV domain from YtvA (# 1–127) fused to the kinase domain of FixL (# 258–505), i.e. it derives its helical linker from FixL. We therefore first composed the triple-fusion protein C9, consisting of the LOV domain of YtvA (# 1–127), the linker helix of FixL (# 259–281) and the histidine kinase- plus response regulator domain from Sln1 (# 567–1221). Phosphorylation assays, however, showed that this construct did not display any measurable kinase activity (Additional file 1: Table S1). Two constructs were then designed, to conserve the sequence around the DIT motif of YtvA, and to expand it to the DITKQ motif. Accordingly, C10 was designed, and also C11, with the deletion of one amino acid downstream of the DITKQ motif (Fig. 2a). The TKQ motif was identified in the Sln1 histidine kinase too and therefore the YtvA sequence was linked to the kinase domain with optimal conservation of this domain and the coiled/coil structure. Of these two constructs, indeed C11 shows light-activation of kinase activity in the auto-phosphorylation assay (Additional file 1: Table S1). However, as the most important in vivo test is best carried out with a kinase of which the activity is decreased upon illumination, construct C11 was not used in the studies of the shuttling of HOG1; it may, however, be of interest for future studies of gene activation in *S. cerevisiae* (c.f. Fig. 3).

### The two signal-transduction pathways emerging from Sln1: shuttling of Ypd1

The introductory figure of this report (Fig. 1) does not make an explicit statement on the issue of whether or not also Ypd1 would act in signal transfer to nuclear components by active shuttling between the two compartments. The available experimental evidence suggests that it does not (Lu et al. 2003), although alternative mechanisms for relay of the Ypd ~ P signal to the nucleus have not been proposed (yet). In relation to this it is relevant to note that we too have tried to observe nuclear accumulation of Ypd1, fused to GFP, with fluorescence microscopy, and could not observe significant nuclear accumulation of this fusion protein either (compare to (Lu et al. 2003)), under conditions that the salt stress gave a very clear response for the HOG1 (GFP-fusion) protein.

### Signal transduction in the Sln1 system and the number of signal-transducing molecules

Beyond the difference in light sensitivity—the main purpose of the experiment—of the two strains reported on in Fig. 3, it is clear that the one with the truncated Sln1 fusion protein shows considerably higher activity in the dark than the unperturbed wild type system. Two possible underlying differences can explain this latter aspect: (i) a higher intrinsic kinase activity of the LOV::Sln1 fusion construct than the authentic Sln1 kinase and (ii) a higher expression level of the fusion kinase. As the kinase is not expressed from its natural promoter, but from the ACT1 promoter, a rather strong, mostly constitutive (but glucose repressible) promotor (Wenzel et al. 1995; Planta et al. 1999) we think that the fusion kinase may be present at higher concentration than Sln1. Nevertheless, a higher intrinsic activity may also play a role as a similar activation has also been observed in some bacterial two-component kinases (Szurmant et al. 2008; Verhamme 2002).

Absolute numbers, and by inference concentrations, of the molecular components of a signal transduction chain are important, particularly in the two-component systems, e.g. because most kinases in the absence of their cognate signal, display considerable phosphatase activity. The approximately 10- to 100-fold molar excess of response regulator over kinase in most bacterial two-component systems testifies to this (e.g. (Yoshida et al. 2002; Wayne et al. 2010)). The results presented in Additional file 1: Fig. S2 show that both in the natural response system to osmotic stress and in the light response, mediated by the hybrid kinase, the HOG1/kinase molar ratio is of crucial importance too. If the concentration of the HOG1::GFP reporter protein is increased from its basal level (in cells that presumably contain only a single copy of the expression plasmid) to more than four to fivefold higher, the signal transduction system seems oversaturated with HOG1, and a response to both stimuli is no longer visible. The unperturbed Sln1 signal transduction system functions with 656 and 6780 molecules per cell of Sln1 kinase and the HOG1 transducer, respectively (Ghaemmaghami et al. 2003). This corresponds to ~ 25 nM and 0.5 μM, respectively in non-stimulated cells. Analysis of the average cellular concentration of the HOG1::GFP fusion protein with fluorescence-correlation microscopy (M. Hink et al., unpublished observation; for methodology see (Maeder et al. 2007)) suggests that its abundance—at the basal, pre-stimulus, level—is 0.15 (± 0.06; n = 41) μM, i.e. slightly lower but still comparable to that of the HOG1 protein in the wild type, in spite of the differences in promoters used. These results suggest that the concentration of the HOG1 protein in the Sln1 signal transduction pathway is such that overexpression of HOG1 above physiological levels will make the Sln1 signal transduction pathway non-functional.

The results shown in Fig. 5 and Additional file 1: Fig. S2 suggest that the on-dynamics of the light response is slower than that of the osmostress response. If so, this may have several causes, like a lower degree of kinase modulation by light, or a suboptimal expression ratio of the proteins composing the light-responsive signal transduction pathway. Furthermore, the open bars in Fig. 5, panel a, do seem to show a very slight remaining stimulation of HOG1 accumulation in the nucleus upon stressing the strain that expresses the light-sensitive, truncated variant of Sln1. This can be explained by weak spill-over of signals from the osmostress-responsive Sho1 system of *S. cerevisiae* into the Sln1 system at the level of the Ssk1 MAP kinase pathway (Hao et al. 2007).

The hybrid kinase described in this study is an excellent candidate for future studies on quantitation of the consequences of e.g. localized kinase activation in the cytoplasm, for the dynamics and amplitude of the overall cellular response. This will allow further fine-tuning, e.g. with respect to the role of (anomalous) cytoplasmic diffusion, of systems biology models developed to describe the osmo/stress response in *S. cerevisiae* (Uschner and Klipp 2014; Dexter et al. 2015). Various super-resolution microscopy techniques are available to facilitate such experiments (e.g. Small and Parthasarathy 2014)). Also the use of specific subcellular localization tags and/or interaction domains can be exploited for this (Schierling and Pingoud 2012).

## Additional file

**Additional file 1.** Additional tables and figures

## Abbreviations

GFP: green fluorescent protein
LOV: light oxygen voltage
LED: light emitting diodes
