# Supplementary material for: Design, characterization and in vivo functioning of a light-dependent histidine protein kinase in the yeast Saccharomyces cerevisiae
Source: AMB Express. 2018 Apr 2;8:53. doi: 10.1186/s13568-018-0582-7 (PMC5880792; doi:10.1186/s13568-018-0582-7)
Supplement: Supplementary file 1 — Additional file 1. Additional tables and figures [file 13568_2018_582_MOESM1_ESM.docx]

**Journal: AMB Express**

**Title: Design, characterization and in vivo functioning of a light-dependent histidine protein kinase in the yeast *Saccharomyces cerevisiae***

**Authors: Aleksandra Bury^1^ and Klaas J. Hellingwerf^1^***

**Affiliations: ^1^: Molecular Microbial Physiology Group, Swammerdam Institute for Life Sciences, University of Amsterdam, Amsterdam, The Netherlands**

*: corresponding author at: Molecular Microbial Physiology Group, Swammerdam Institute for Life Sciences, University of Amsterdam, Science Park 904, 1098 XH Amsterdam, The Netherlands, tel.: +31-20-5257055; e-mail: [K.J.Hellingwerf@uva.nl](mailto:K.J.Hellingwerf@uva.nl)

**Additional data**

Table S1. Details of the design of a series of LOV::Sln1 histidine kinase fusion proteins.

C1 contains the LOV domain and the Jα linker from YtvA from *B. subtilis* and the histidine kinase domain from Sln1 from *S. cerevisae*, while C2 is composed of only the LOV-domain part from YtvA, connected to the coiled/coil sequence preceding the histidine kinase of Sln1. The key DIT motif of YtvA is positioned between #125 – 127 in the Jα linker. The phosphorylatable histidine of the Sln1 protein histidine kinase domain is located immediately downstream of the linker helix. In variant C6 an extra amino acid was inserted in the 549 position (↓549H). ^*^: this sequence from the hepta-helical signature domain of the coiled-coil structure of FixL was inserted in-between the sequences of LOV-domain of YtvA and the kinase domain of Sln1p. n.d.: not determined; n.a.: not available.

| Construct name | Numbers of amino acid from YtvA | Numbers of amino acid from Sln1 | Initial rate of phosphorylation in the dark  (nmolP/g protein/minute) | Initial rate of phosphorylation in the light  (nmolP/g protein/minute) | Ratio | Standard deviation |
| --- | --- | --- | --- | --- | --- | --- |
| C1 | YtvA (1-146) | Sln1HKR1 (567-1221) | 0.18 | 0.08 | 2.25 | 0.35 |
| C2 | YtvA (1-127) | Sln1HKR1 (538-1221) | 0.08 | 0.08 | 1 | 0.23 |
| C5 | YtvA (1-127) | Sln1HKR1 (536-1221) | 0.03 | 0.03 | 1 | 0.10 |
| C6 | YtvA (1-127) | Sln1HKR1 (536-1221) ↓549H | 0.08 | 0.09 | 0.9 | 0.05 |
| C8 | YtvA (1-132) | Sln1HKR1 (540-1221) | 0.01 | 0.01 | 1 | 0.12 |
| C9 | YtvA (1-127)  FixL (259-281)^*^ | Sln1HKR1 (567-1221) | n.d | n.d | n.d | n.d |
| C10 | YtvA (1-129) | Sln1HKR1 (553-1221) | 0.20 | n.a. | -- | -- |
| C11 | YtvA (1-129) | Sln1HKR1 (554-1221) | 0.11 | 0.21 | 0.5 | n.d |

Table S2: Strains and plasmids used in this investigation

| Strain or plasmid | Characteristics | Source or reference |
| --- | --- | --- |
| ***Saccharomyces cerevisiae* BY4741** | | |
| ∆YLR113W | MATα HOG1::kanMX,  his3D200, leu2D1, ura3-52 trp1D63 lys2D201 can^R^ cyh^R^ | G. Smits, University of Amsterdam ^1^ |
| ∆YIL147C, ∆ ∆YLR113W | MATα, sln::clonNAT, HOG1::kanMX his3D200, leu2D1, ura3-52 trp1D63 lys2D201 can^R^ cyh^R^; pRS325ActC1LEU, pRS416-HOG1::GFP | This study |
| ∆ YLR113W – GFP | MATα, HOG1::kanMX,  his3D200, leu2D1, ura3-52 trp1D63 lys2D201 can^R^ cyh^R^, pRS416-HOG1::GFP | This study |
| ∆YIL147C | MATα, sln::clonNAT, his3D200, leu2D1, ura3-52 trp1D63 lys2D201 can^R^ cyh^R^; pRS325ActC1LEU, pRS314-OCH1(-336 to =26)-lacZ | This study |
| BY4741 | MATa, his3Δ1, leu2Δ0, met15Δ0, ura3Δ0pRS314-OCH1(-336 to =26)-lacZ | This study |
| ***Escherichia coli*** | | |
| Xl1-Blue | Cloning host | Agilent technologies, [Santa Clara, California](https://en.wikipedia.org/wiki/Santa_Clara,_California), [United States](https://en.wikipedia.org/wiki/United_States) |
| M15 (pREP4) | Overexpression host | Qiagen, [Hilden, Duitsland](https://www.google.nl/search?client=firefox-b&dcr=0&q=Hilden+Duitsland&stick=H4sIAAAAAAAAAOPgE-LUz9U3sDQ2z7JQAjON401yk7S0spOt9POL0hPzMqsSSzLz81A4VhmpiSmFpYlFJalFxQBsGJzXRAAAAA&sa=X&ved=0ahUKEwi3uqX7qsHZAhXPLFAKHV9jB-cQmxMIpQEoATAQ) |
| **Plasmids** | | |
| pRS325ActC1LEU | pRS325II derivate, ACT1 promoter, cyc1 terminator, kan^R^ | This study |
| pRS416-HOG1::GFP |  | P. Silver (Ferrigno, Posas et al. 1998) |
| pJL1416 | pRS314-OCH1(-336 to +26)-*lacZ* | J.S. Fassler (Lu, Deschenes et al. 2003) |
| pAB009 | pQE30 overexpression vector for the C1 protein | This study^*^ |
| pAB010 | pQE30 overexpression vector for the C2 protein | This study^*^ |
| pAB025 | pQE30 overexpression vector for the C5protein | This study^*^ |
| pAB026 | pQE30 overexpression vector for the C6 protein | This study^*^ |
| pAB014 | pQE30 overexpression vector for the C8 protein | This study^*^ |
| pAB022 | pQE30 overexpression vector for the C9 protein | This study^*^ |
| pAB023 | pQE30 overexpression vector for the C10 protein | This study^*^ |
| pAB024 | pQE30 overexpression vector for the C11 protein | This study^*^ |
| pAB011 | pQE30 overexpression vector for the Ypd1 protein | This study |

^1^: ∆YLR113W is the HOG1-deletion strain from the EUROSCARF collection, which has used the BY4741 *S. cerevisiae* strain (MATa, *his3*Δ1, *leu*2Δ0, *met*15Δ0, *ura*3Δ0) as its wild type.

^*^: For more details about C1-C11 see Table S1.

**Fig. S1.** Kinetics of the incorporation [^32^Pi] into the C1 histidine kinase protein. [γ-^32^P]-ATP was used as the phosphoryl group donor. Reaction mixtures were incubated either in the presence of light (open symbols, dashed lines) or in the dark (filled symbols, full lines). Analysis of the amount of phosphorylated protein was carried out as described in Materials and Methods.

a.

b.

**Fig. S2**: Image analysis of individual *S. cerevisiae* cells exposed to signal transfer that induces nuclear shuttling of a Ypd1-GFP fusion protein Representation of the distribution in the cells of the HOG1::GFP protein against the intensity of the HOG1::GFP fluorescence in the yeast cells before (T0) and at two time points after salt stress with 0.4 M NaCl (final concentration), panel a, and before and after exposure of the cells to blue light (200 µEinstein·m^2^·s^-1^ intensity, 450 nm LED light), panel b, after 2 and 5 minutes. In – fluorescence intensity in the nucleus, Ic – fluorescence intensity in the cytoplasm.
